# The Effects of *Lactobacillus*-Fermented Feed Derived from Tibetan Black Pigs and Changbai Mountain Wild Boars on the Growth Performance, Meat Quality, and Immune Function of Duroc × Landrace × Yorkshire (DLY) Finishing Pigs

**DOI:** 10.3390/microorganisms14071562

**Published:** 2026-07-17

**Authors:** Hao Yu, Lei Wang, Guofang Wu, Boyu Lu, Xuan Luo, Yinghao Zhang, Xinhui Luo, Liping Cheng

**Affiliations:** 1 College of Animal Husbandry and Veterinary Sciences, Qinghai University, Xining 810016, China; 15908257578@163.com (H.Y.); qhuwuguofang@126.com (G.W.); lby0002024@163.com (B.L.); cauluoxuan@163.com (X.L.); 19853953177@163.com (Y.Z.); 18336066852@163.com (X.L.); 15693884476@163.com (L.C.); 2 Qinghai Provincial Plateau Livestock Genetic Resources Protection and Innovative Utilization Laboratory, Xining 810016, China; 3 Qinghai-Tibet Plateau Key Laboratory of Animal Genetics and Breeding, Ministry of Agriculture and Rural Affairs, Xining 810016, China

**Keywords:** lactic acid bacteria-fermented feed, DLY finishing pigs, growth performance, meat quality, immune and antioxidant properties, gut microbiota

## Abstract

To develop green feed additives suitable for antibiotic-free livestock farming on the Qinghai–Tibet Plateau, this study used *Lactobacillus salivarius* TB2.1, isolated from Tibetan black pigs, and *Lactobacillus agilis* CW13.2, isolated from wild boars in the Changbai Mountains, to prepare fermented feed. The study investigated the effects of these additives on the growth performance, slaughter performance, meat quality, immune and antioxidant functions, and gut microbiota composition of Duroc × Landrace × Large White crossbred finishing pigs (DLY finishing pigs). The experiment included 72 finishing pigs with an average body weight of 54.07 ± 1.02 kg, which were randomly divided into three groups, each with four replicates of 6 pigs. The control group (CK) was fed a basal diet, one treatment group (TB) was fed a diet consisting of 50% basal diet plus 50% *Lactobacillus salivarius* TB2.1-fermented feed, and the other treatment group (CW) was fed a diet consisting of 50% basal diet plus 50% *Lactobacillus agilis* CW13.2-fermented feed. The pre-feeding period lasted 3 days, and the experimental period lasted 57 days. The results showed that, compared with the CK group, (1) the CW and TB groups had significantly higher levels of crude protein, lactic acid, acetic acid, and propionic acid in the feed (*p* < 0.05), and significantly lower levels of crude fiber, neutral detergent fiber, acid detergent fiber, ammonium nitrogen, and pH (*p* < 0.05); (2) in the CW and TB groups, final body weight, average daily weight gain, and average daily feed intake were significantly higher (*p* < 0.001), while the feed-to-gain ratio was significantly lower (*p* < 0.01), with the CW group performing better than the TB group; (3) in both the CW and TB groups, carcass weight, eye muscle area, and meat redness significantly increased (*p* < 0.001), while backfat thickness and shear force significantly decreased (*p* < 0.001); (4) in both the CW and TB groups, serum immunoglobulin G, interleukin-2, and superoxide dismutase levels significantly increased (*p* < 0.05), malondialdehyde levels were significantly reduced in the CW group (*p* < 0.05), and catalase activity was significantly increased in the TB group (*p* < 0.05); (5) the relative abundance of *Escherichia* spp. in the cecum was significantly reduced in both the CW and TB groups (*p* < 0.05), while beneficial bacteria such as *Lactobacillus* spp. were significantly enriched (*p* < 0.05). In summary, feed fermented with *Lactobacillus salivarius* TB2.1 and *Lactobacillus agilis* CW13.2 can effectively improve the production performance of finishing pigs, enhance meat quality, strengthen immune and antioxidant capacities, and optimize the gut microbiota structure. Among these, CW13.2 was more effective in promoting weight gain, while TB2.1 was more effective in regulating protein and lipid metabolism. Fermented feed produced with both strains shows promising application prospects and can provide high-quality microbial strains and technical support for antibiotic-free swine farming on the Qinghai–Tibet Plateau.

## 1. Introduction

The long-term misuse of antibiotics in animal feed not only leads to an imbalance in the gut microbiota and a weakened immune system in livestock and poultry, but also triggers the spread of bacterial resistance and drug residues in animal products, severely hindering the green and sustainable development of the livestock industry [1]. Following the full implementation of China’s antibiotic ban in July 2020, the development of safe, efficient, and environmentally friendly antibiotic-free feed additives has become a key focus of livestock research [2]. Probiotics can improve animal growth performance and gut health by regulating the balance of the gut microbiota, secreting antibacterial substances such as lactic acid and bacteriocins, enhancing host immune function, and improving feed nutrient conversion efficiency; they are therefore ideal additives for replacing antibiotics [3,4]. Lactic acid bacteria (LAB), as the most widely used probiotics, can degrade antinutritional factors such as crude fiber and phytic acid, convert macromolecular proteins into small-molecule substances such as oligopeptides and free amino acids, and simultaneously improve feed palatability. They play a significant role in enhancing the growth performance of livestock and poultry, improving meat quality, and maintaining gut health [5,6,7].

In recent years, lactic acid bacteria probiotics have been widely used in animal husbandry for disease prevention and control as well as health management due to their high safety profile and consistent efficacy. They play a key role in maintaining the balance of the gut microbiome, regulating the immune system, and promoting host health [8,9,10,11]. As demand for high-quality livestock products continues to rise, the value of lactic acid bacteria in swine production has attracted increasing attention [12]. Numerous studies have shown that feed-added probiotics can positively regulate the production and health of pigs at different growth stages [13]. The addition of lactic acid bacteria can significantly improve piglets’ average daily weight gain, average daily feed intake, and feed conversion ratio, while also increasing small intestinal villus height. During the weaning stage, adding *Lactobacillus salivarius*, *Lactobacillus plantarum*, or *Lactobacillus rhamnosus* to the diet can significantly improve growth performance, intestinal morphology, and immune function [14,15,16]. The combined supplementation of lactic acid bacteria and bifidobacteria from Tibetan pigs also has good growth-promoting and immune-modulating effects [17], and inactivated lactic acid bacteria preparations also help improve serum immune markers and the intestinal microbiota [18]. During the growing pig phase, liquid fermented compound probiotic feed can reduce antinutritional factors, increase daily weight gain, and improve meat quality [19]. During the finishing stage, host-specific, multi-strain lactic acid bacteria probiotics can increase the final pH and reduce cooking losses, thereby improving pork quality [20]. Lactic acid bacteria derived from pigs offer unique advantages in the development of feed additives due to their excellent acid and bile salt tolerance, antibacterial activity, and adaptability to the host [21]. Research has shown that strains such as *Lactobacillus* amylopectin, derived from Tibetan pigs, have been proven to possess strong probiotic properties [22]. Therefore, screening and utilizing superior *Lactobacillus* strains derived from hosts to produce fermented feed has become a key focus in research on and development of pig feed additives in the context of antibiotic-free livestock farming.

LAB refers to the core gut microbiota of animals; probiotics derived from the same species as the host are more likely to colonize the gut and exert their beneficial effects [23,24]. The Qinghai–Tibet Plateau features unique environmental conditions—including high altitude, low oxygen levels, low temperatures, and significant diurnal temperature fluctuations—which have given rise to highly stress-tolerant local pig breeds such as the Tibetan black pig and the Eight-Brow pig. Long-term natural selection has shaped the gut microbiota of these breeds into a unique structure adapted to extreme environments, harboring a wealth of lactic acid bacteria that are highly stress-tolerant and possess excellent probiotic properties. Research indicates that high-altitude environments can alter the composition of the Tibetan pig’s gut microbiota—8210 genes have been detected in the guts of high-altitude Tibetan pigs, and their microbial community structure differs from that of low-altitude, pen-raised pigs; furthermore, 73.47% of the species-level genomes belong to newly discovered species [25]. Fibrobacterota and Elusimicrobia are significantly enriched in the intestines of Tibetan pigs and are involved in the synthesis of short-chain fatty acids such as acetic acid, butyric acid, and propionic acid, as well as 20 essential amino acids and various B vitamins, which help the pigs adapt to extreme environments such as high-altitude hypoxia and intense ultraviolet radiation [25]. In addition, the abundance of microbial communities in the intestines of Tibetan pigs associated with short-chain fatty acid synthesis and the digestion of cellulose and hemicellulose increased significantly [26], and these communities were capable of degrading dietary fiber [27]. Metagenomic analysis further revealed that more than 13,000 carbohydrate-degrading genes have been identified in the gut microbiome of Tibetan pigs, distributed across five phyla, including Firmicutes and Bacteroidetes [28]. Therefore, compared with commercial strains, lactic acid bacteria isolated from local high-altitude pigs are better adapted to the farming environment of the Qinghai–Tibet Plateau and the intestinal microbiome of pigs, and exhibit greater stability in application. Previous studies have successfully isolated lactic acid bacteria strains from Tibetan pig feces that possess excellent probiotic characteristics [22], such as rapid growth, the production of various active enzymes that break down carbohydrates, and strong adhesion to intestinal epithelial cells, confirming the development potential of lactic acid bacteria isolated from local high-altitude pigs as probiotic preparations.

Based on this, this study utilized *Lactobacillus salivarius* TB2.1 and *Lactobacillus agilis* CW13.2—which were previously screened by our research group (characterized by rapid growth, strong acid production, bile salt tolerance, broad-spectrum antibacterial activity, safety, non-pathogenicity, and cellulase activity)—to conduct fermentation feed preparation and fattening pig feeding trials. The study investigates the effects of fermentation feeds containing lactic acid bacteria derived from Tibetan black pigs and wild boars on the growth, meat quality, and immune function of DLY finishing pigs. The objective is to screen for superior probiotic lactic acid bacteria suitable for the plateau environment, thereby providing a theoretical basis and microbial strain support for antibiotic-free livestock farming on the Qinghai–Tibet Plateau and the development of lactic acid bacteria feed additives.

## 2. Materials and Methods

### 2.1. Experimental Materials

The test strains were *Lactobacillus salivarius* TB2.1 and *Lactobacillus agilis* CW13.2. TB2.1 was isolated from fresh feces of Tibetan black pigs at the Zhenwang Breeding Cooperative in Ledu District, Haidong City, Qinghai Province, while CW13.2 was isolated from fresh feces of Changbai Mountain wild boars at the Mutuo Wangsheng Specialized Breeding Cooperative in Qinghai Province. One gram of each fresh fecal sample was collected and subjected to a 10-fold serial dilution in sterile saline. A 100 μL aliquot of the appropriate dilution was evenly spread onto MRS-CaCO_3_ identification agar plates (MRS solid medium supplemented with 1% CaCO_3_) and incubated at 37 °C for 24 h. Single colonies with distinct calcium-dissolving zones were selected. After three rounds of streaking for purification, single colonies were inoculated into MRS liquid medium and incubated at 37 °C for 24 h. The purified bacterial suspension was mixed with 30% sterile glycerol in a 1:1 (*v*/*v*) ratio, aliquoted into cryovials, and stored long-term at −80 °C for future use. Identification via 16S rRNA gene sequencing (primers 27F/1492R) revealed that TB2.1 is *Lactobacillus salivarius* and CW13.2 is *Lactobacillus agilis*.

After thawing the aforementioned cryopreserved strains, they were inoculated into MRS liquid medium and incubated at 37 °C for 24 h to prepare a seed culture. Transfer the seed culture to fresh MRS liquid medium at a 5% (*v*/*v*) inoculation rate for expansion, incubate at 37 °C for 24 h, and obtain a bacterial suspension for fermentation. As determined by plate counting, the viable cell count of TB2.1 was 5.1 × 10^9^ CFU/mL, and that of CW13.2 was 1.6 × 10^10^ CFU/mL.

Determine the feed and water volumes in a 1:1 ratio, adjust the feed moisture content to approximately 45–50%, and evenly spray the prepared lactic acid bacteria suspension onto the feed at an inoculation rate of 5% (*v*/*w*). Mix the feed while spraying, and then use a mixer to thoroughly blend for 5 min to ensure even distribution of the bacterial suspension throughout the feed. Place the mixed fermented feed into polyethylene plastic bags, press out the air, seal the bags, and allow them to undergo anaerobic fermentation at room temperature for 10 days.

Samples were collected before and after fermentation, and sensory evaluation was conducted to observe the color, odor, and texture of the fermented feed in order to assess the fermentation process and degree of maturity. On the 10th day of fermentation, the fermented feed was yellowish-brown in color, had a strong sour aroma, showed no signs of mold or off-odors, and had a loose texture that was not sticky to the touch. Additionally, a 500 g sample was collected on the 10th day of fermentation and stored at −80 °C for subsequent nutritional analysis to determine whether fermentation was complete.

The baseline diet was formulated according to the nutritional requirements of finishing pigs; ingredients and nutritional components are shown in Table 1.

### 2.2. Experimental Design

The experiment selected 72 DLY finishing pigs with an average weight of 54.07 ± 1.02 kg, which were randomly divided into 3 groups, each with 4 replicates of 6 pigs. The control group (CK) was fed the basal diet; the first experimental group (TB) was fed a diet consisting of 50% basal diet + 50% *Lactobacillus salivarius* TB2.1-fermented feed (on a dry matter basis); and the second experimental group (CW) was fed a diet consisting of 50% basal diet + 50% *Lactobacillus agilis* CW13.2-fermented feed (on a dry matter basis). The pre-feeding period lasted 3 days, and the experimental period lasted 57 days. During the trial, pigs had free access to water and were fed once in the morning and once in the evening each day. Immunization, disinfection, and hygiene procedures were carried out according to standard protocols. The trial was conducted at Qinghai Fuyuan Agriculture and Animal Husbandry Technology Co., Ltd. (Xining, China), which provided both the pigs and the basal diet.

### 2.3. Test Parameters and Methods

#### 2.3.1. Feed Quality

We collected 500 g of feed samples before and after fermentation and stored them in a −80 °C freezer for later use. Chemical composition analysis was conducted by Shanghai Enzyme-Linked Biotechnology Co., Ltd. (Shanghai, China), including dry matter (DM), crude protein (CP), crude fiber (CF), neutral detergent fiber (NDF), acid detergent fiber (ADF), soluble sugar (SS), and starch (ST), determined in accordance with GB/T 6435-2014, GB/T 6432-2018, GB/T 6434-2022, GB/T 20806-2006, NY/T 1459-2022, GB/T 37493-2019, and GB/T 42491-2023, respectively. Ammonia nitrogen (NH_3_-N) was determined using the salicylic acid–hypochlorite spectrophotometric method. Lactic acid (LA), acetic acid (AA), propionic acid (PA), and butyric acid (BA) were determined using high-performance liquid chromatography.

#### 2.3.2. Growth Performance

During the experiment, feed was provided at regular intervals each day, and the feed troughs were kept full throughout the day to ensure ad libitum feeding and drinking. Each morning before feeding, the remaining feed was cleaned and weighed (after removing feces and impurities), and converted to air-dry weight to calculate the average daily feed intake. Fasting body weights were measured before morning feeding on days 1 and 57 of the trial to calculate the average daily gain (ADG), average daily feed intake (ADFI), and feed-to-gain ratio (F/G).

Average daily gain = weight gain/number of trial days;

Average daily feed intake = total feed intake/number of trial days;

Feed-to-gain ratio = average daily feed intake/average daily gain.

#### 2.3.3. Slaughter Performance

Measure the carcass weight, eye muscle area, and backfat thickness in accordance with NY/T 825-2004, “Technical Specifications for the Determination of Carcass Characteristics of Lean-Type Pigs.”

#### 2.3.4. Meat Quality

In accordance with NY/T 1333-2007, “Determination of Meat Quality of Livestock and Poultry,” the following parameters were measured: pH at 45 min, meat color, shear force, cooked meat yield, moisture loss, and pH at 24 h.

#### 2.3.5. Serum Indicators

At the conclusion of the feeding trial, 6 pigs were randomly selected from each group, and venous blood was collected in the early morning on an empty stomach using vacuum blood collection tubes containing sodium heparin as an anticoagulant. After collection, the blood samples were allowed to stand for 30 min, and then centrifuged at 3000 r/min for 10 min. The serum was transferred to 1.5 mL centrifuge tubes and stored at −20 °C; serum parameter testing was outsourced to Lilai Biotechnology (Harbin, China).

(1) Serum biochemical parameters: Glucose (GLU), total cholesterol (TC), triglycerides (TG), and total protein (TP) were measured using a veterinary biochemical analyzer (manufactured by Shenzhen Mindray Medical Electronics Co., Ltd., Shenzhen, China, model BS-460).
(2) Serum immunological parameters: Immunoglobulin A (IgA), immunoglobulin G (IgG), immunoglobulin M (IgM), Tumor Necrosis Factor-alpha (TNF-α), interleukin-2 (IL-2), interleukin-6 (IL-6), and interleukin-10 (IL-10) were measured using commercial porcine-derived ELISA kits; all procedures were performed according to the kit instructions.
(3) Serum antioxidant parameters: Superoxide dismutase (SOD), malondialdehyde (MDA), catalase (CAT), and Total Antioxidant Capacity (T-AOC) were measured using commercially available porcine-derived ELISA kits; all procedures were performed according to the kit instructions.

#### 2.3.6. Gut Microbiota

From each group, six pigs were randomly selected for slaughter. The contents of the jejunum and cecum were collected into 5 mL cryovials, stored in liquid nitrogen, and sent to Shenzhen Weike Meng Technology Group Co., Ltd. (Shenzhen, China) for 16S rDNA gene sequencing. Sequencing was performed using the Illumina PE250 platform (Illumina, San Diego, CA, USA). Quality control of all raw sequences from all samples was conducted using the DADA2 plugin [29] within the QIIME 2 (version 2023.5) software [30], and species annotation information was obtained by aligning the sequences with the SILVA database [31] (using the qiime feature-classifier classify-sklearn method).

### 2.4. Data Statistics and Analysis

After organizing the data in Excel 2019, a one-way analysis of variance (one-way ANOVA, LSD) was performed using the One-ANOVA program. Results are presented as “mean ± standard deviation” (mean ± SD). A *p*-value of <0.05 indicates a significant difference, <0.01 indicates a highly significant difference, and >0.05 indicates no significant difference. 16S rDNA sequencing data were visualized using Wekemo Bioincloud (https://www.bioincloud.tech) [5].

## 3. Results

### 3.1. Changes in Nutritional Composition of Feed Before and After Lactobacillus Fermentation

As shown in Table 2, compared with the CK group, the DM and CP contents in the CW and TB groups were both extremely significantly higher (*p* < 0.001), while the CF, NDF, and ADF contents were all extremely significantly lower (*p* < 0.001). Regarding carbohydrate components, the SS and ST contents in the TB group were extremely significantly higher than those in the CK group (*p* < 0.001), while the SS and ST contents in the CW group were extremely significantly lower than those in the CK group (*p* < 0.001). Regarding fermentation quality, NH_3_-N content in both the CW and TB groups was extremely significantly lower than that in the CK group (*p* < 0.001); LA, AA, and PA contents were all extremely significantly higher than those in the CK group (*p* < 0.001); BA content was significantly lower than that in the CK group (*p* < 0.05); and pH values were all extremely significantly lower than those in the CK group (*p* < 0.001).

Comparisons among the treatment groups showed that the TB group had extremely significantly higher DM, CP, SS, ST, LA, AA, and PA contents than the CW group (*p* < 0.001), while its NH_3_-N content was extremely significantly lower than that of the CW group (*p* < 0.001); regarding NDF and ADF content, the CW group was extremely significantly lower than the TB group (*p* < 0.001), while there was no significant difference in CF content between the two groups (*p* > 0.05); there was no significant difference in BA content or pH between the two groups (*p* > 0.05).

### 3.2. Effects of Lactobacillus-Fermented Feed on the Growth Performance of DLY Finishing Pigs

As shown in Table 3, there were no significant differences in initial body weight among the groups (*p* > 0.05). Compared with the CK group, the CW and TB groups showed extremely significant increases in final body weight, ADFI, and ADG (*p* < 0.001), and extremely significant decreases in F/G (*p* < 0.01).

Comparisons among the experimental groups showed that the final body weight, ADFI, and ADG in the CW group were all extremely significantly higher than those in the TB group (*p* < 0.001), while the F/G ratio was extremely significantly lower than that in the TB group (*p* < 0.01).

### 3.3. Effects of Lactobacillus-Fermented Feed on Slaughter Performance of DLY Finishing Pigs

As shown in Table 4, compared with the CK group, the CW group exhibited extremely significant increases in body weight, backfat thickness, and eye muscle area (*p* < 0.001), while there was no significant difference in backfat weight (*p* > 0.05); in the TB group, there was no significant difference in body weight (*p* > 0.05), but backfat thickness and eye muscle area were both extremely significantly higher (*p* < 0.001), and there was no significant difference in lard weight (*p* > 0.05).

Comparisons among the experimental groups showed that body weight in the CW group was extremely significantly higher than that in the TB group (*p* < 0.001), while there were no significant differences in backfat thickness or eye muscle area between the two groups (*p* > 0.05); there were no significant differences in backfat weight among the three groups (*p* > 0.05).

### 3.4. Effects of Lactobacillus-Fermented Feed on Meat Quality in DLY Finishing Pigs

As shown in Table 5, compared with the CK group, both the CW and TB groups exhibited a highly significant increase in meat color score (*p* < 0.001) and a highly significant decrease in shear force (*p* < 0.001); the CW group showed a highly significant increase in pH at 24 h (*p* < 0.01), while the TB group showed no significant difference in pH at 24 h (*p* > 0.05); pH at 45 min was significantly lower in the CW group (*p* < 0.05), while there was no significant difference in the TB group (*p* > 0.05); and there were no significant differences among the three groups in terms of lightness (L), yellowness (b), cooked meat yield, or water loss rate (*p* > 0.05).

Comparisons among the treatment groups showed that the meat color score in the TB group was extremely significantly lower than that in the CW group (*p* < 0.001), and the shear force was extremely significantly lower than that in the CW group (*p* < 0.001); regarding pH at 45 min, the TB group was significantly higher than the CW group (*p* < 0.05), while there was no significant difference between the two groups for pH at 24 h (*p* > 0.05).

### 3.5. Effects of Lactobacillus-Fermented Feed on Serum Biochemical Parameters in DLY Finishing Pigs

As shown in Table 6, compared with the CK group, the TG and TP levels in the TB group were both significantly higher (*p* < 0.05), while there were no significant differences in TG and TP levels between the TB and CW groups (*p* > 0.05); there were no significant differences in GLU and TC levels among the three groups (*p* > 0.05).

Comparisons among the experimental groups showed that the TG and TP levels in the TB group were both significantly higher than those in the CW group (*p* < 0.05), while there were no significant differences in GLU and TC levels between the two groups (*p* > 0.05).

### 3.6. Effects of Lactobacillus-Fermented Feed on Serum Immune Parameters in DLY Finishing Pigs

As shown in Table 7, compared with the CK group, both the CW and TB groups exhibited extremely significant increases in IgG levels (*p* < 0.001) and significant increases in IL-2 levels (*p* < 0.01); the CW group showed a significant increase in IL-10 levels (*p* < 0.01), while the TB group showed no significant difference in IL-10 levels (*p* > 0.05); IL-6 levels in the TB group showed an upward trend but did not reach statistical significance (*p* = 0.052), while the CW group showed no significant difference (*p* > 0.05); and there were no significant differences in IgA, IgM, or TNF-α levels among the three groups (*p* > 0.05).

Comparisons between the experimental groups showed that IL-10 levels in the CW group were significantly higher than those in the TB group (*p* < 0.01), while there were no significant differences between the two groups for the other immune markers (*p* > 0.05).

### 3.7. Effects of Lactobacillus-Fermented Feed on Serum Antioxidant Parameters in DLY Finishing Pigs

As shown in Table 8, compared with the CK group, SOD activity was extremely significantly higher in both the CW and TB groups (*p* < 0.001); MDA levels were significantly lower in the CW group (*p* < 0.05), while there was no significant difference in MDA levels in the TB group (*p* > 0.05); CAT activity was significantly higher in the TB group (*p* < 0.05), while there was no significant difference in CAT activity in the CW group (*p* > 0.05); and there were no significant differences in T-AOC among the three groups (*p* > 0.05).

Comparisons among the experimental groups showed that SOD activity in the CW group was significantly higher than that in the TB group (*p* < 0.05), and MDA content was significantly lower than that in the TB group (*p* < 0.05); however, there were no significant differences in CAT activity or T-AOC between the two groups (*p* > 0.05).

### 3.8. Effects of Lactobacillus-Fermented Feed on Gut Microbiota Composition in DLY Finishing Pigs

#### 3.8.1. Effects of Lactic Acid Bacteria-Fermented Feed on the Alpha Diversity of the Gut Microbiota in DLY Finishing Pigs

**Figure 1:**
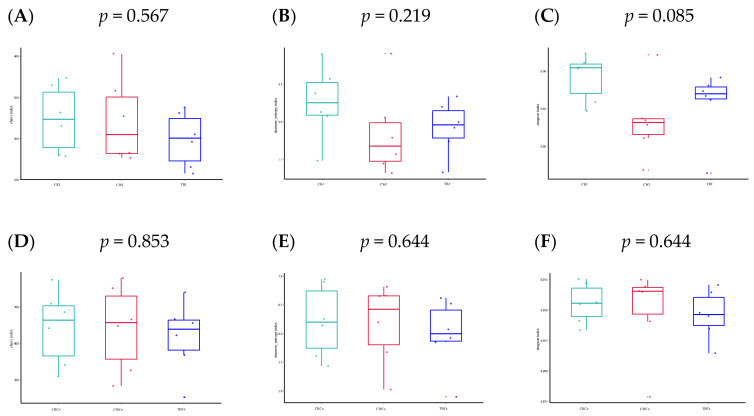
Alpha diversity analysis of jejunal and cecal flora. (**A**) is the jejunal Chao1 index, (**B**) is the jejunal Shannon index, (**C**) is the jejunal Simpson index, (**D**) is the cecum Chao1 index, (**E**) is the cecum Shannon index and (**F**) is the cecum Simpson index.

As shown in Figure 1A–C, compared with the CK group, there were no significant differences (*p* > 0.05) in the Chao-1 index, Shannon index, and Simpson index of the jejunum in the TB and CW groups (*p* = 0.567, *p* = 0.219, *p* = 0.085); as shown in Figure 1D–F, compared with the CK group, there were no significant differences in the Chao-1 index, Shannon index, and Simpson index (*p* = 0.853, *p* = 0.644, *p* = 0.644) in the cecum of the TB and CW groups (*p* > 0.05), indicating that lactic acid bacteria-fermented feed had no significant effect on alpha diversity in the jejunum and cecum.

#### 3.8.2. Effects of Lactic Acid Bacteria-Fermented Feed on the Beta Diversity of the Gut Microbiota in DLY Finishing Pigs

**Figure 2:**
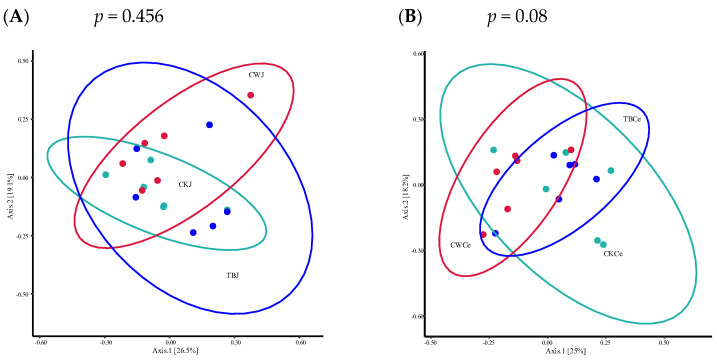
Beta diversity analysis of jejunal (**A**) and cecal (**B**) flora.

Principal Coordinate Analysis (PCoA) based on the Bray–Curtis distance algorithm revealed changes in gut microbiota beta diversity among different groups. As shown in Figure 2A, the contribution values of the first and second principal components for jejunal samples were 26.5% and 19.1%, respectively, with a *p*-value of 0.456; as shown in Figure 2B, the contribution values of the principal components for cecal samples were 25% and 18.2%, respectively, with a *p*-value of 0.08, indicating that the microbial community composition was similar across the three groups, with no significant differences.

#### 3.8.3. Effects of Lactic Acid Bacteria-Fermented Feed on the Composition of the Gut Microbiota in DLY Finishing Pigs

**Figure 3:**
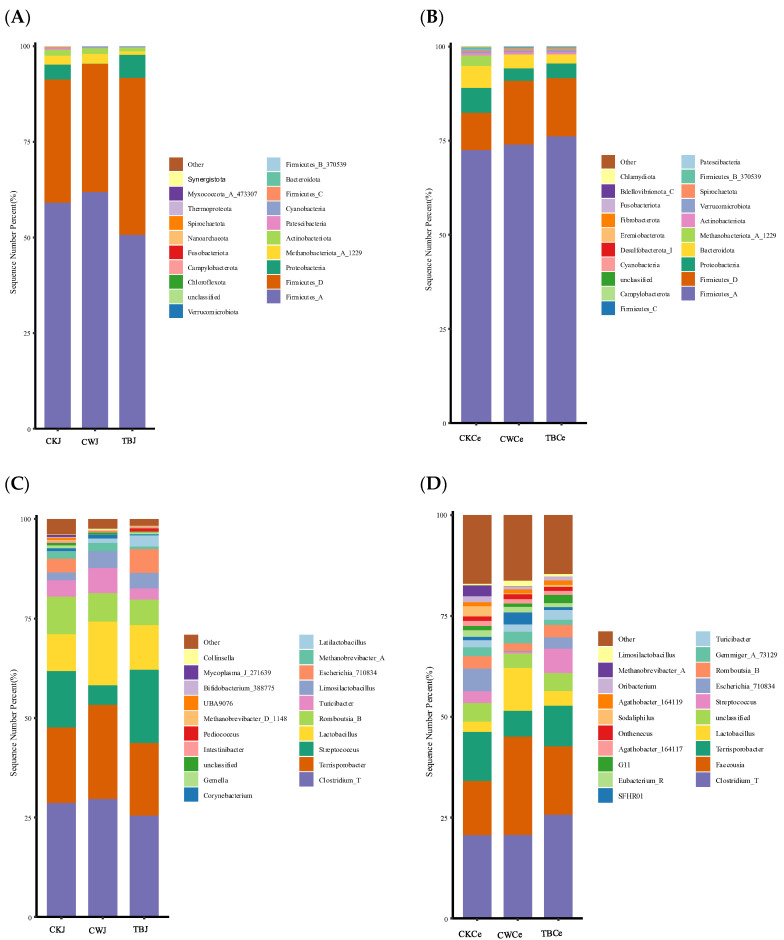
Species composition at the phylum and genus levels of the jejunal and cecal flora. (**A**) is the level of jejunal phyla, (**B**) is the level of cecum phyla, (**C**) is the level of jejunal genera, and (**D**) is the level of cecum genera.

At the phylum level, as shown in Figure 3A, the dominant phyla in the jejunum of each group were Firmicutes_A, Proteobacteria, Bacteroidota, and Methanobacteriota_A, with Firmicutes_A having the highest relative abundance, followed by Proteobacteria. The relative abundance of Firmicutes was higher in the CW and TB groups than in the control group, while the relative abundance of Proteobacteria was similar across all groups. The relative abundances of Bacteroidota and Methanobacteriota were low across all groups and showed no significant differences. Figure 3B shows that the dominant phyla in the cecum were also primarily Firmicutes and Proteobacteria. The relative abundance of Firmicutes was higher in the CW and TB groups than in the control group, while the relative abundance of Proteobacteria was similar across all groups. The relative abundance of Bacteroidota was low in all groups, and there were no significant differences in the relative abundances of the remaining phyla across groups.

At the genus level, as shown in Figure 3C, the dominant genera in the jejunum across all groups were *Lactobacillus*, *Streptococcus*, *Clostridium_T,* and *Terrisporobacter*, with *Lactobacillus* having the highest relative abundance, followed by *Streptococcus*; the relative abundance of *Lactobacillus* was higher in the CW and TB groups than in the control group; the relative abundance of *Pediococcus* was higher in the TB group than in the CW group and the control group; the relative abundance of *Latilactobacillus* was higher in both the CW and TB groups than in the control group; and the relative abundance of *Escherichia_710834* was lower in the CW group than in the control group and the TB group. As shown in Figure 3, the dominant genera in the cecum were *Clostridium_T*, *Faecousia*, *Terrisporobacter*, and *Lactobacillus*, with *Clostridium_T* having the highest relative abundance, followed by *Faecousia*. In the CW group, the relative abundances of *Lactobacillus* and *Faecousia* were both higher than those in the control and TB groups. The relative abundance of *Escherichia_710834* was lower in both the CW and TB groups compared to the control group, with a more pronounced decrease in the CW group; the relative abundances of the remaining genera were low across all groups and showed no significant differences.

#### 3.8.4. Analysis of Differences in the Gut Microbiome of DLY Finishing Pigs Fed *Lactobacillus*-Fermented Feed

**Figure 4:**
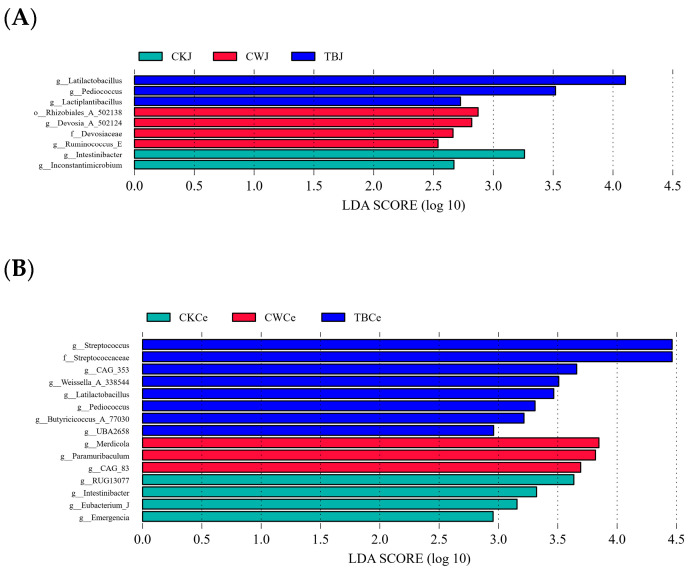
Differentially enriched bacterial genera in the jejunum (**A**) and cecum (**B**) based on LEfSe analysis (LDA > 2, *p* < 0.05). The LEfSe analysis employed a pairwise comparison strategy across multiple groups; the figure shows the bacterial genera significantly enriched in each treatment group relative to the other two groups. Red represents bacterial genera significantly enriched in the control group (CK), green represents those significantly enriched in the CW group, and blue represents those significantly enriched in the TB group; the LDA values reflect the contribution of each differential microbial community to the intergroup differences (effect size).

Based on LEfSe analysis (LDA > 2, *p* < 0.05), we screened for differentially enriched bacterial communities in each group relative to the other two groups using pairwise comparisons across multiple groups (Figure 4). As shown in Figure 4A, in the jejunum, the genera *Intestinibacter* (LDA = 2.5) and *Inconstantimicrobium* (LDA = 2.5) were significantly enriched in the CK group; in the CW group, the significantly enriched genera were *Devosia_A_502124* (LDA = 2.8) and *Ruminococcus_E* (LDA = 2.5); and in the TB group, the significantly enriched genera were *Latilactobacillus* (LDA = 3.8), *Pediococcus* (LDA = 3.8), and *Lactiplantibacillus* (LDA = 2.8).

As shown in Figure 4B, in the cecum, the bacterial genera significantly enriched in the CK group were *RUG13077* (LDA = 3.5), *Intestinibacter* (LDA = 3.5), *Eubacterium_J* (LDA = 3.5), and *Emergencia* (LDA = 3.5); in the CW group, the significantly enriched genera were *Merdicola* (LDA = 3.5), *Paramuribaculum* (LDA = 3.5), and *CAG_83* (LDA = 3.5); and in the TB group, the significantly enriched genera were *Streptococcus* (LDA = 3.8–4.0), *CAG_353* (LDA = 3.5), *Weissella_A_338544* (LDA = 3.5), *Latilactobacillus* (LDA = 3.5), *Pediococcus* (LDA = 3.5), *Butyricicoccus_A_77030* (LDA = 3.5), and *UBA2658* (LDA = 3.5).

## 4. Discussion

### 4.1. Effects of Lactobacillus-Fermented Feed Quality

Lactic acid bacteria fermentation can effectively improve feed quality. By utilizing substances such as SS for growth and reproduction, it rapidly produces organic acids such as LA and AA, thereby lowering the feed’s pH. At the same time, the rapid acidification process effectively inhibits protein degradation, thus reducing the production of NH_3_-N in the fermentation products [32]. Research by Shi et al. [33] showed that after solid-state fermentation of a corn–soybean meal feed mixture using Bacillus subtilis and Enterococcus faecalis, the levels of CP, trichloroacetic acid-soluble protein, ash, and total phosphorus significantly increased, while NDF, hemicellulose, and phytic acid phosphorus content significantly decreased. Soybean antigenic protein was substantially degraded, and in vitro dry matter and crude protein digestibility significantly improved. A study by Chen et al. [34] showed that after solid-state fermentation of soybean meal with Bacillus subtilis and *Lactobacillus plantarum*, the levels of crude protein, free amino acids, and lactic acid significantly increased, while crude fiber and phytic acid content decreased by 45.9% and 48.1%, respectively. This is similar to the results of the present study, in which the contents of CP, LA, AA, and PA all increased significantly, while CF, NDF, ADF, NH_3_-N, BA, and pH all decreased significantly, while the SS and ST contents in the CW group both decreased significantly, whereas those in the TB group increased significantly. It is speculated that TB2.1 may have secreted certain hydrolases, such as cellulase, which can hydrolyze cellulose into small-molecule sugars such as cellobiose; these hydrolyzed small-molecule sugars may have been detected as soluble sugars, thereby increasing the soluble sugar content in the feed. At the same time, the BA content in the fermented feed decreased significantly compared to the unfermented feed, although the LA and AA contents increased significantly. This phenomenon may be related to competitive microbial metabolism during fermentation. First, the inoculated *Lactobacillus salivarius* TB2.1 and *Lactobacillus agilis* CW13.2 proliferate rapidly under anaerobic conditions, extensively utilizing soluble carbohydrates (SS) as a carbon source and prioritizing metabolic pathways toward homolactic or heterolactic fermentation. This results in the massive production of LA and AA, reducing the conversion of acetyl-CoA to BA and consequently lowering the BA content. A study by Zhu et al. [35] showed that when the pH falls below 5.7, the fermentation products of Clostridium tyrobutyricum shift from being predominantly butyric acid to being predominantly lactic acid and acetic acid; this shift is associated with changes in the activity of butyryl-CoA phosphatase and lactate dehydrogenase. Furthermore, when the pH drops to around 5.0 during fermentation, most butyrate-producing bacteria (such as those in the genus Clostridium) are extremely sensitive to acidic environments. Thylin et al. [36] found that the inhibitory effect of lactic acid on Clostridium tyrobutyricum is significantly enhanced under low-pH conditions, and that rapidly lowering the pH is key to inhibiting Clostridium growth. Furthermore, the NH_3_-N content decreased significantly during fermentation, suggesting that fermented feed may inhibit protein degradation. A study by Lü et al. [37] showed that adding more than 2 g-N/L of NH_3_-N significantly inhibits butyric acid production from protein substrates. Therefore, the significant decrease in NH_3_-N content observed in this experiment may have inhibited protein degradation, thereby limiting the supply of substrates for butyric acid production via amino acid redox fermentation pathways.

### 4.2. Effects of Lactobacillus-Fermented Feed on Growth Performance, Slaughter Performance, and Meat Quality in DLY Finishing Pigs

Growth performance and slaughter performance are important parameters for evaluating an animal’s growth status and fattening efficiency, including ADG, eye muscle area, and backfat thickness. The findings of Rezazadeh et al. [38] in calves are consistent with this, indicating that fermented soybean meal can improve feed utilization efficiency in weaned calves to enhance production performance. Jones et al. [39] found that adding 6% fermented soybean meal to the diet significantly increased ADG and ADFI in growing–finishing pigs. In this study, compared with the CK group, the final body weight, ADG, and ADFI were all significantly higher in the CW and TB groups; the F/G ratio was significantly lower in the CW group and showed a decreasing trend in the TB group; carcass weight was significantly higher in the CW group and showed an increasing trend in the TB group; and backfat thickness and eye muscle area were both significantly reduced in the CW and TB groups. This may be due to the breakdown and elimination of antinutritional factors in the feed by probiotics during the fermentation process, thereby facilitating digestion and absorption in the animals. Meat color primarily reflects the hue of the meat [40], shear force reflects tenderness [41], and cooked meat yield and moisture loss rate are important indicators of meat water-holding capacity [42]. In this study, the redness of meat in the TB and CW groups was significantly higher than that in the control group, while the yellowness of meat in the CW group and the cooked meat yield in the TB group both showed an upward trend. Studies by Feng et al. [43] and Hao et al. [44] have shown that fermented feed can effectively increase cooked meat yield, enhance meat redness, and improve palatability. These findings indicate that probiotic-fermented feed can improve pork quality, which is consistent with the results of this experiment.

### 4.3. Effects of Lactobacillus-Fermented Feed on Serum Biochemical, Immunological, and Antioxidant Parameters in DLY Finishing Pigs

Biochemical parameters of blood reflect the body’s health status and changes resulting from internal and external factors [45]. Serum levels of TP, GLU, TC, and TG all reflect the body’s protein, energy, and fat metabolism. The results of this experiment show that serum TG and TP levels in the TB group were significantly higher than those in the control and CW groups. Elevated TP levels suggest that fermented feed may promote protein synthesis; elevated serum TG levels facilitate fat synthesis and accumulation in the body, thereby promoting animal fattening [46]. Serum levels of IgG, IgA, and IgM are important indicators of an animal’s immune capacity and constitute components of the immune system [47]. As core effector molecules of the humoral immune response, immunoglobulins can specifically recognize pathogens; their levels reflect the intensity of the humoral immune response and the body’s ability to clear pathogens [48]. The expression levels of cytokines such as IL-6, IL-10, and TNF-α are correlated with the health status of animals and play a crucial role in the immune system; they work in concert to maintain the body’s immune balance and homeostasis [49]. Relevant studies have shown that adding an appropriate proportion of fermented feed to the diet can effectively improve the aforementioned immune indicators, replace antibiotics in performing anti-inflammatory and other functions, and enhance the health of weaned piglets [50,51]. Elevated serum levels of IgA and IgG enhance the animal’s immune response [52]. In this experiment, serum IgG and IL-2 levels in the experimental group were significantly higher than those in the control group; IL-6 levels in the TB group and IL-10 levels in the CW group were both significantly elevated, while other indicators showed no significant changes. These results suggest that fermented feed may stimulate the immune system through lactic acid bacteria metabolites, which in turn is associated with increased levels of factors such as immunoglobulins and interleukins. However, the underlying mechanisms require further validation through subsequent cellular or molecular biological experiments.

Serum antioxidant markers reflect an animal’s defensive capacity and the stability of its internal environment; oxygen free radicals produced by animal metabolism can cause cellular damage and harm animal health [53]. SOD is an important antioxidant enzyme in living organisms; it reflects the degree of intracellular oxidative stress and serves as the primary scavenger of reactive oxygen species [54]. CAT is an enzyme that catalyzes the decomposition of hydrogen peroxide into water and oxygen, protecting cells from oxidative damage. MDA reflects the degree of lipid oxidation and is associated with oxidative stress-induced damage [55]. Czech et al. [56] found that adding 6% fermented rapeseed meal to the diet of weaned piglets increased serum SOD and CAT activity, reduced MDA levels, and improved the redox status of the weaned piglets. In this study, serum SOD levels were significantly increased in both the CW and TB groups; MDA levels in the CW group were significantly lower than those in the CK group, while the TB group showed a decreasing trend, though the difference was not significant. CAT levels in the TB group were significantly higher than those in the CK group, while the CW group showed a trend toward increase; there were no significant differences in T-AOC levels among the groups. The two strains exhibited differences in their regulation of oxidative stress markers: feeding with CW13.2 significantly reduced serum MDA, focusing on the prevention of lipid oxidation damage; TB2.1, on the other hand, primarily and significantly enhanced CAT activity, focusing on the activation of the endogenous antioxidant enzyme system. The two strains follow distinct antioxidant regulatory pathways: the CW strain tends to focus on clearing lipid peroxides, while the TB strain primarily enhances the secretion of antioxidant enzymes. This difference may be related to the metabolic characteristics developed by the two strains as a result of being isolated from their hosts and undergoing long-term acclimatization to the plateau environment. The results indicate that feeding animals probiotic-fermented feed may enhance their antioxidant capacity and improve animal health [57].

### 4.4. Effects of Lactobacillus-Fermented Feed on the Gut Microbiota of DLY Finishing Pigs

The gut is the primary site for digestion and absorption in animals. A healthy gut microbiota not only participates in physiological activities such as the immune response and nutrient absorption, but also inhibits the colonization of pathogenic bacteria [58], thereby maintaining the animal’s health [59,60]. In this study, there were no significant differences in alpha and beta diversity of the jejunal and cecal microbiota among the three groups. This suggests that while probiotic-fermented feed increases the abundance of beneficial bacteria, it may not disrupt the stability of the gut microbiota, thereby helping to maintain gut microbiota balance—an important prerequisite for the long-term safe use of feed additives. At the phylum level, the dominant microbial communities in the jejunum and cecum of the three groups of finishing pigs were all Firmicutes, Proteobacteria, Bacteroidota, and Methanobacteriota, which is consistent with the findings of related studies on the core microbial communities of the porcine gut [61]. Firmicutes are dominant bacteria involved in the microbial degradation of cellulose and the improvement of feed energy utilization [62], while Bacteroidota are primarily involved in carbohydrate metabolism and nutrient absorption [63]. Proteobacteria typically include most intestinal pathogenic bacteria, such as *Escherichia coli*; some strains (such as enterohemorrhagic *E. coli*) may cause diseases such as intestinal inflammation and diarrhea [64,65].

Based on the results of this study, further analysis indicates that lactic acid bacteria-fermented feed improves the availability of intestinal nutrients by increasing the crude protein (CP) content and reducing the levels of crude fiber and antinutritional factors, which may in turn optimize the gut microbiota. Compared with the CK group, the relative abundance of *Escherichia* spp. in the cecum was significantly reduced in both the CW and TB groups, while beneficial bacteria such as *Lactobacillus* spp. were significantly enriched. This indicates that *Lactobacillus salivarius* TB2.1, isolated from Tibetan black pigs, and *Lactobacillus agilis* CW13.2, isolated from wild boars in the Changbai Mountains, can maintain intestinal health by regulating the gut microbiota structure and inhibiting the colonization of harmful bacteria. Improved gut health may further enhance the animals’ rate of nutrient digestion and absorption, and may also boost their immunoglobulin levels and antioxidant capacity, ultimately contributing to improved growth performance, slaughter performance, and meat quality in finishing pigs. Overall, there is a consistent trend between the feeding of lactic acid bacteria-fermented feed and changes in gut microbiota composition (enrichment of *Lactobacillus* and reduction in Escherichia), elevated immune markers (IgG, IL-2), and improved antioxidant function (SOD, CAT), suggesting that it may exert a positive influence on the health of finishing pigs through a “microbiota regulation–host response” pathway. However, the molecular mechanisms underlying these associations still require further elucidation through metabolomics, transcriptomics, and other analytical methods. It is worth noting that the two lactic acid bacteria strains used in this study were isolated from the intestines of Tibetan black pigs on the Qinghai–Tibet Plateau (*Lactobacillus salivarius* TB2.1) and wild boars in the Changbai Mountains (*Lactobacillus agilis* CW13.2). Tibetan black pigs have long lived in high-altitude (2400–3800 m) environments characterized by low oxygen levels, intense ultraviolet radiation, and significant diurnal temperature fluctuations; as a result of prolonged natural selection, their gut microbiota has developed unique stress-tolerant characteristics [25]. Studies have shown that high-altitude environments can significantly alter the composition of the gut microbiota in Tibetan pigs, leading to a significant increase in the abundance of fiber-degrading bacteria and their carbohydrate metabolism capacity [27]. In this study, the TB2.1 strain isolated from Tibetan black pigs was found to modulate cellulase activity, acid production, and protein and lipid metabolism, which may be related to its host’s long-term adaptation to a high-fiber, low-nutrient grazing diet. These findings suggest that probiotics of high-altitude origin may exhibit greater environmental adaptability in the Qinghai–Tibet Plateau farming environment, thereby helping to increase the abundance of beneficial bacteria and maintain the balance of the gut microbiota.

### 4.5. Analysis of Differences in Fermentation Performance Among Different Lactobacillus Strains

This study found that the two lactic acid bacteria strains, CW13.2 and TB2.1, exhibited strain-specific differences in fermentation quality and host effects. In terms of fermentation quality, the TB group had significantly higher CP, SS, and ST contents than the CW group, while the CW group was more effective at reducing NDF and ADF. This difference may be related to differences in the enzymatic profiles of the two strains: TB2.1 possesses higher cellulase activity, which hydrolyzes cellulose into small-molecule sugars such as cellobiose, thereby increasing soluble sugar content; CW13.2, on the other hand, tends to degrade fiber, resulting in a greater reduction in fiber components. Different *Lactobacillus* strains exhibit significant differentiation in substrate utilization and metabolic products due to differences in their carbohydrate-active enzyme (CAZyme) gene profiles. Studies have found that a strain’s CAZyme composition is related to its isolation environment; for example, strains derived from kefir grains possess more enzymes associated with cellulose metabolism, indicating that the habitat environment shapes the strain-specific carbohydrate utilization capabilities [66]. Further research has shown that the presence of genes alone cannot fully explain metabolic differences; the regulatory context of genes is equally important—even if a strain carries a gene cluster involved in the metabolism of a specific sugar, that metabolic pathway may still fail to be expressed if there are transcriptional repressors downstream of it [67]. This explains why the two bacterial strains, although both capable of producing enzymes, utilize substrates differently. Furthermore, the utilization of oligosaccharides by lactic acid bacteria exhibits strain specificity and substrate specificity, and genomic analysis can reveal CAZymes specific to different substrates [68]. In terms of host effects, the CW group outperformed the TB group in promoting growth, reducing the F/G ratio, and increasing carcass weight, suggesting that CW13.2 may be more effective at converting fermentation products into body tissue. Conversely, the TB group was more effective at improving serum TG and TP levels, indicating that TB2.1 may be more effective at regulating protein and lipid metabolism in the body. In terms of antioxidant function, the CW group showed a more significant reduction in MDA, while the TB group demonstrated a greater increase in CAT, suggesting that the two bacterial strains differ in their mechanisms for alleviating oxidative stress [69].

The specific differences between the two strains may be related to their host origins and ecological adaptations: *Lactobacillus salivarius* TB2.1 was isolated from the gut of high-altitude Tibetan black pigs, whose hosts have long been adapted to a high-fiber, low-nutrient grazing diet; this strain may therefore possess stronger polysaccharide degradation and metabolic regulation capabilities. *Lactobacillus agilis* CW13.2 was isolated from wild boars in the Changbai Mountains; its metabolic characteristics are more oriented toward the efficient utilization of fermentation products to promote host growth. Studies have shown that lactic acid bacteria strains from different isolation sources exhibit significant differences in functional genes and CAZyme composition, and these differences are closely related to the strain’s isolation environment [21]. CAZyme profiles of plant-derived isolates are typically better adapted to the degradation of complex carbohydrates, whereas animal-derived strains possess a broader gene pool for bacteriocin synthesis [70]. Probiotic strains derived from the host itself often exhibit stronger adaptability to the host [71,72]. This finding provides a reference basis for subsequent targeted screening and formulation of strains based on specific production objectives (emphasizing either weight gain or meat quality).

## 5. Conclusions

Fermented feed prepared using *Lactobacillus salivarius* TB2.1 from Tibetan black pigs and *Lactobacillus agilis* CW13.2 from wild boars in the Changbai Mountains can optimize the nutritional composition of feed. Feeding these two types of fermented feed had significant effects on both weight gain and health: they increased the average daily weight gain of finishing pigs, enhanced immune and antioxidant functions, and inhibited the colonization of harmful gut bacteria. Of the two strains, TB2.1, derived from the Tibetan black pig, demonstrated superior performance in regulating protein metabolism, highlighting the unique application value of lactic acid bacteria from high-altitude pig breeds in the Qinghai–Tibet Plateau farming environment. In summary, these two types of fermented feed can provide high-quality microbial resources and experimental references for antibiotic-free, eco-friendly pig farming on the Qinghai–Tibet Plateau; however, their underlying mechanisms require further study.

## Figures and Tables

**Table 1:** Base diet composition and nutrient content (%).

Ingredients	Composition	Nutrient Levels	
Corn (%)	71.00	DE (MJ/kg)	13.46
Soybean meal (%)	13.00	CP (%)	14.28
Bran (%)	8.00	Lys (%)	0.96
Premix (%)	8.00	Ca (%)	0.72
Total	100.00	P (%)	0.53

Note: The premix provided the following per kilogram of the diets: VA 44,000 IU, VD3 7000 IU, VE 200 mg, VK3 15 mg, VB2 35 mg, VB5 88 mg, Cu 120 mg, Fe 770 mg, Zn 2800 mg, Mn 300 mg, I 5 mg, and Se 2 mg. DE was calculated using Tables of Feed Composition and Nutritional Values in China (34rd Edition 2023), while the others were measured values.

**Table 2:** Comparison of nutrient composition before and after feed fermentation.

Items	Control Group (CK)	Test Group (CW)	Test Group (TB)	*p*-Value
DM/%	43.62 ± 0.13 ^c^	45.98 ± 0.17 ^b^	50.21 ± 0.12 ^a^	<0.001
CP/%	14.28 ± 0.07 ^c^	16.12 ± 0.04 ^b^	17.90 ± 0.14 ^a^	<0.001
CF/%	23.20 ± 0.13 ^a^	19.30 ± 0.23 ^b^	19.00 ± 0.57 ^b^	<0.001
NDF/%	57.16 ± 0.56 ^a^	48.78 ± 0.45 ^c^	50.72 ± 0.24 ^b^	<0.001
ADF/%	24.39 ± 0.22 ^a^	20.14 ± 0.08 ^c^	22.50 ± 0.28 ^b^	<0.001
SS (mg/g)	16.32 ± 0.18 ^b^	13.87 ± 0.05 ^c^	19.62 ± 0.35 ^a^	<0.001
ST (mg/g)	146.30 ± 0.17 ^b^	131.90 ± 0.46 ^c^	173.90 ± 0.85 ^a^	<0.001
NH_3_-N (μg/g)	5982.00 ± 47.34 ^a^	4879.00 ± 45.61 ^b^	3998.00 ± 56.58 ^c^	<0.001
LA (mg/g)	22.34 ± 0.19 ^c^	45.82 ± 0.11 ^b^	48.85 ± 0.58 ^a^	<0.001
AA (mg/g)	0.74 ± 0.05 ^c^	6.77 ± 0.11 ^b^	8.88 ± 0.04 ^a^	<0.001
PA (mg/g)	0.23 ± 0.02 ^c^	0.60 ± 0.02 ^b^	0.72 ± 0.01 ^a^	<0.001
BA (mg/g)	2.87 ± 0.02 ^a^	1.39 ± 0.06 ^b^	1.55 ± 0.04 ^b^	0.025
pH	6.26 ± 0.06 ^a^	4.95 ± 0.07 ^b^	5.02 ± 0.03 ^b^	<0.001

Note: In the same row, values with no letter or same-letter superscripts are not significantly different (*p* > 0.05), while those with different small-letter superscripts are significantly different (*p* < 0.05), and those with different capital-letter superscripts are significantly different (*p* < 0.01). The same applies below.

**Table 3:** Effects of fermented feed on the growth performance of DLY finishing pigs.

Items	Control Group (CK)	Test Group (CW)	Test Group (TB)	*p*-Value
Initial weight (IW), kg	54.01 ± 0.91 ^a^	54.16 ± 1.47 ^a^	54.30 ± 0.71 ^a^	0.903
Final weight (FW), kg	86.00 ± 1.41 ^c^	96.83 ± 0.82 ^a^	90.00 ± 0.22 ^b^	<0.001
ADFI, kg/d	2.49 ± 0.01 ^c^	2.92 ± 0.05 ^a^	2.74 ± 0.04 ^b^	<0.001
ADG, kg/d	0.56 ± 0.03 ^c^	0.74 ± 0.02 ^a^	0.62 ± 0.01 ^b^	<0.001
F/G	4.45 ± 0.26 ^a^	3.92 ± 0.21 ^b^	4.37 ± 0.11 ^a^	0.001

**Table 4:** Effect of fermented feeds on slaughter performance of DLY finishing pigs.

Items	Control Group (CK)	Test Group (CW)	Test Group (TB)	*p*-Value
Carcass weight (CW), kg	62.46 ± 1.84 ^b^	68.73 ± 1.16 ^a^	64.18 ± 0.47 ^b^	<0.001
Backfat thickness (BFT), mm	16.53 ± 0.64 ^b^	20.79 ± 1.28 ^a^	19.37 ± 0.52 ^a^	<0.001
Leaf fat weight (LFW), kg	0.60 ± 0.06	0.62 ± 0.06	0.63 ± 0.04	0.733
Loin eye area (LEA), cm^2^	24.41 ± 1.37 ^b^	27.10 ± 0.75 ^a^	28.60 ± 0.77 ^a^	<0.001

**Table 5:** Effects of fermented feed on meat quality in DLY finishing pigs.

Items		Control Group (CK)	Test Group (CW)	Test Group (TB)	*p*-Value
Meat color	Brightness, L*	41.37 ± 0.79 ^a^	41.22 ± 0.5 ^a^	41.14 ± 0.52 ^a^	0.805
	Redness, a*	4.47 ± 0.19 ^c^	5.92 ± 0.16 ^a^	5.22 ± 0.14 ^b^	<0.001
	Yellowness, b*	5.84 ± 0.16 ^a^	5.90 ± 0.32 ^a^	5.81 ± 0.09 ^a^	0.771
pH_45 min_		6.43 ± 0.1 ^ab^	6.30 ± 0.06 ^b^	6.50 ± 0.14 ^a^	0.015
pH_24 h_		5.67 ± 0.06 ^b^	5.96 ± 0.21 ^a^	5.62 ± 0.12 ^b^	0.003
Shear force, N		39.70 ± 0.68 ^a^	34.73 ± 1.01 ^b^	32.53 ± 0.92 ^c^	<0.001
Cooked meat rate, %		67.07 ± 1.65 ^a^	65.82 ± 2.11 ^a^	68.20 ± 2.26 ^a^	0.160
Water loss rate, %		5.50 ± 0.26 ^a^	5.41 ± 0.59 ^a^	4.89 ± 1.02 ^a^	0.291

**Table 6:** Effects of fermented feed on serum biochemical parameters in DLY finishing pigs.

Items	Control Group (CK)	Test Group (CW)	Test Group (TB)	*p*-Value
Glu (mmol/L)	5.96 ± 1.02 ^a^	5.54 ± 1.11 ^a^	5.56 ± 0.41 ^a^	0.671
TC (mmol/L)	2.39 ± 0.19 ^a^	2.51 ± 0.21 ^a^	2.58 ± 0.13 ^a^	0.227
TG (mmol/L)	0.35 ± 0.03 ^b^	0.36 ± 0.02 ^b^	0.52 ± 0.05 ^a^	<0.001
TP (mmol/L)	73.92 ± 2.45 ^b^	73.98 ± 2.3 ^b^	78.58 ± 4.43 ^a^	0.036

**Table 7:** Effects of fermented feed on serum immune parameters in DLY finishing pigs.

Items	Control Group (CK)	Test Group (CW)	Test Group (TB)	*p*-Value
IgA (μg/mL)	10.64 ± 0.98 ^a^	10.67 ± 0.46 ^a^	11.31 ± 0.16 ^a^	0.149
IgG (mg/mL)	2.24 ± 0.06 ^b^	2.97 ± 0.09 ^a^	2.99 ± 0.20 ^a^	<0.001
IgM (mg/mL)	1.20 ± 0.08 ^a^	1.29 ± 0.09 ^a^	1.21 ± 0.09 ^a^	0.181
IL-2 (pg/mL)	51.88 ± 1.85 ^b^	56.31 ± 1.92 ^a^	54.22 ± 1.62 ^a^	0.003
IL-6 (pg/mL)	115.69 ± 3.25 ^b^	115.62 ± 4.56 ^b^	120.56 ± 2.93 ^a^	0.052
IL-10 (pg/mL)	29.79 ± 0.97 ^b^	33.66 ± 1.92 a	30.77 ± 1.64 ^b^	0.002
TNF-α (pg/mL)	8.63 ± 0.72 ^a^	8.78 ± 0.85 ^a^	9.64 ± 0.85 ^a^	0.099

**Table 8:** Effects of fermented feed on serum antioxidant parameters in DLY finishing pigs.

Items	Control Group (CK)	Test Group (CW)	Test Group (TB)	*p*-Value
SOD (U/mL)	336.64 ± 3.68 ^c^	357.34 ± 7.49 ^a^	347.22 ± 8.54 ^b^	<0.001
MDA (nmol/mL)	2.85 ± 0.26 ^a^	2.50 ± 0.14 ^b^	2.69 ± 0.22 ^ab^	0.034
CAT (U/mL)	5.00 ± 0.16 ^b^	5.15 ± 0.15 ^ab^	5.30 ± 0.13 ^a^	0.012
T-AOC (mM)	0.34 ± 0.04 ^a^	0.34 ± 0.05 ^a^	0.33 ± 0.04 ^a^	0.944

## Data Availability

The raw sequencing data, chromatographic analysis data, and experimental sample materials generated in this study contain proprietary preliminary research data relevant to subsequent series of studies, in-depth validation of mechanisms, translation of research findings, and patent applications; therefore, the raw research data will not be made public at this stage. For non-commercial academic research purposes, requests for access to the relevant data may be submitted via formal email to the corresponding author.

## References

[1] Shivaramaiah S., Pumford N.R., Morgan M.J., Wolfenden R.E., Wolfenden A.D., Torres-Rodríguez A., Hargis B.M., Téllez G. (2011). Evaluation of Bacillus species as potential candidates for direct-fed microbials in commercial poultry. Poult. Sci..

[2] Zhu X.Y., Joerger R.D. (2003). Composition of microbiota in content and mucus from cecae of broiler chickens as measured by fluorescent in situ hybridization with group-specific, 16S rRNA-targeted oligonucleotide probes. Poult. Sci..

[3] Reuter G. (2001). Probiotics--possibilities and limitations of their application in food, animal feed, and in pharmaceutical preparations for men and animals. Berl. Munch. Tierarztl. Wochenschr..

[4] Li N.N., Wang Q., Wang Y., Sun A.J., Lin Y.W., Jin Y., Li X.B. (2018). Oral Probiotics Ameliorate the Behavioral Deficits Induced by Chronic Mild Stress in Mice via the Gut Microbiota-Inflammation Axis. Front. Behav. Neurosci..

[5] Canani R.B., Cirillo P., Terrin G., Cesarano L., Spagnuolo M.I., De Vincenzo A., Albano F., Passariello A., De Marco G., Manguso F. (2007). Probiotics for treatment of acute diarrhoea in children: Randomised clinical trial of five different preparations. BMJ-Br. Med. J..

[6] Hill C., Guarner F., Reid G., Gibson G.R., Merenstein D.J., Pot B., Morelli L., Canani R.B., Flint H.J., Salminen S. (2014). Expert consensus document. The International Scientific Association for Probiotics and Prebiotics consensus statement on the scope and appropriate use of the term probiotic. Nat. Rev. Gastroenterol. Hepatol..

[7] Chaucheyras-Durand F., Durand H. (2010). Probiotics in animal nutrition and health. Benef. Microbes.

[8] Liu G., Tan F.H., Lau S.A., Jaafar M.H., Chung F.Y., Azzam G., Liong M.T., Li Y. (2022). Lactic acid bacteria feeding reversed the malformed eye structures and ameliorated gut microbiota profiles of Drosophila melanogaster Alzheimer’s disease model. J. Appl. Microbiol..

[9] Brashears M.M., Amezquita A., Jaroni D. (2005). Lactic acid bacteria and their uses in animal feeding to improve food safety. Adv. Food Nutr. Res..

[10] Ashraf R., Shah N.P. (2014). Immune system stimulation by probiotic microorganisms. Crit. Rev. Food Sci. Nutr..

[11] Dargahi N., Johnson J., Donkor O., Vasiljevic T., Apostolopoulos V. (2019). Immunomodulatory effects of probiotics: Can they be used to treat allergies and autoimmune diseases?. Maturitas.

[12] Aristimuño Ficoseco C., Mansilla F.I., Maldonado N.C., Miranda H., Nader-Macias M.E.F., Vignolo G.M. (2018). Safety and Growth Optimization of Lactic Acid Bacteria Isolated From Feedlot Cattle for Probiotic Formula Design. Front. Microbiol..

[13] Wang W., Gänzle M. (2019). Toward rational selection criteria for selection of probiotics in pigs. Adv. Appl. Microbiol..

[14] Kinara E., Moturi J., Hosseindoust A., Mun J.Y., Tajudeen H., Ha S.H., Park S.R., Lee S.H., Kim J.S. (2025). Dietary supplementation of *Lactobacillus salivarius* in suckling and weanling piglets modulates intestinal microbiota, morphology and improves growth performance. J. Anim. Sci. Technol..

[15] Kyoung H., Park K.I., Shin S., Ahn J., Nam J., Kang Y., Hwang H., Kim Y., Kim S.W., Jang Y.T. (2026). Probiotic Lactiplantibacillus plantarum improved growth performance of weaned pigs by enhancing intestinal health and modulating immune responses. J. Anim. Sci. Technol..

[16] Shin I., Kang Y., Ahn J., Kim Y., Nam J., Kim K., Kim J.M., Kim H.W., Yang J., Kim Y. (2025). The potential probiotic role of Lacticaseibacillus rhamnosus on growth performance, gut health, and immune responses of weaned pigs. J. Anim. Sci..

[17] He Y., Liang J., Liu Y., Zhou X., Peng C., Long C., Huang P., Feng J., Zhang Z. (2023). Combined supplementation with *Lactobacillus* sp. and Bifidobacterium thermacidophilum isolated from Tibetan pigs improves growth performance, immunity, and microbiota composition in weaned piglets. J. Anim. Sci..

[18] Shu Z., Zhang J., Zhou Q., Peng Y., Huang Y., Zhou Y., Zheng J., Zhao M., Hu C., Lan S. (2024). Effects of inactivated *Lactobacillus rhamnosus* on growth performance, serum indicators, and colonic microbiota and metabolism of weaned piglets. BMC Vet. Res..

[19] Ji M., Rong X., Wu Y., Li H., Zhao X., Zhao Y., Guo X., Cao G., Yang Y., Li B. (2025). Effects of Fermented Liquid Feed with Compound Probiotics on Growth Performance, Meat Quality, and Fecal Microbiota of Growing Pigs. Animals.

[20] Sahatsanon K., Chaweewan K., Sringarm K., Arjin C., Hnokaew P., Satsook A., Saman P., Kim H.W., Patthararangsarith P., Busayakanit P. (2026). Effects of Host-Specific Multi-Lactic Acid Bacterial Probiotics on Performance, Carcass Traits, Meat Quality, and Gut Microbiome in Fattening Pigs. Vet. Sci..

[21] Kavanova K., Kostovova I., Moravkova M., Kubasova T., Crhanova M. (2025). In vitro characterization of lactic acid bacteria and bifidobacteria from wild and domestic pigs: Probiotic potential for post-weaning piglets. BMC Microbiol..

[22] Shen J., Zhang J., Zhao Y., Lin Z., Ji L., Ma X. (2022). Tibetan Pig-Derived Probiotic *Lactobacillus amylovorus* SLZX20-1 Improved Intestinal Function via Producing Enzymes and Regulating Intestinal Microflora. Front. Nutr..

[23] Chen L., Bai X., Wang T., Liu J., Miao X., Zeng B., Li D. (2023). Gut Microbial Diversity Analysis of Different Native Chickens and Screening of Chicken-Derived Probiotics. Animals.

[24] Anisimova E.A., Yarullina D.R. (2019). Antibiotic Resistance of *LACTOBACILLUS* Strains. Curr. Microbiol..

[25] Zhao F., Yang L., Zhang T., Zhuang D., Wu Q., Yu J., Tian C., Zhang Z. (2023). Gut microbiome signatures of extreme environment adaption in Tibetan pig. npj Biofilms Microbiomes.

[26] Liu C., Dan H., Yang Y., Du Y., Hao Z., Chen L., Zhu K., Liu B., Niu L., Zhao Y. (2024). Enhanced immunity: The gut microbiota changes in high-altitude Tibetan pigs compared to Yorkshire pigs. Front. Microbiol..

[27] Yang L., Yao B., Zhang S., Yang Y., Wang G., Pan H., Zeng X., Qiao S. (2025). Division mechanism of labor in Diqing Tibetan Pigs gut microbiota for dietary fiber efficiently utilization. Microbiol. Res..

[28] Bai X., Gu Y., Li D., Li M. (2025). Gut Metagenome Reveals the Microbiome Signatures in Tibetan and Black Pigs. Animals.

[29] Callahan B.J., McMurdie P.J., Rosen M.J., Han A.W., Johnson A.J., Holmes S.P. (2016). DADA2: High-resolution sample inference from Illumina amplicon data. Nat. Methods.

[30] Caporaso J.G., Kuczynski J., Stombaugh J., Bittinger K., Bushman F.D., Costello E.K., Fierer N., Peña A.G., Goodrich J.K., Gordon J.I. (2010). QIIME allows analysis of high-throughput community sequencing data. Nat. Methods.

[31] Quast C., Pruesse E., Yilmaz P., Gerken J., Schweer T., Yarza P., Peplies J., Glöckner F.O. (2013). The SILVA ribosomal RNA gene database project: Improved data processing and web-based tools. Nucleic Acids Res..

[32] Queiroz L.L., Hoffmann C., Lacorte G.A., de Melo Franco B.D., Todorov S.D. (2022). Genomic and functional characterization of bacteriocinogenic lactic acid bacteria isolated from Boza, a traditional cereal-based beverage. Sci. Rep..

[33] Shi C., Zhang Y., Lu Z., Wang Y. (2017). Solid-state fermentation of corn-soybean meal mixed feed with *Bacillus subtilis* and *Enterococcus faecium* for degrading antinutritional factors and enhancing nutritional value. J. Anim. Sci. Biotechnol..

[34] Chen Q., Liu B., Liu G., Shi H., Wang J. (2023). Effect of Bacillus subtilis and *Lactobacillus plantarum* on solid-state fermentation of soybean meal. J. Sci. Food Agric..

[35] Zhu Y., Yang S.T. (2004). Effect of pH on metabolic pathway shift in fermentation of xylose by *Clostridium tyrobutyricum*. J. Biotechnol..

[36] Thylin I., Schuisky P., Lindgren S., Gottschal J.C. (1995). Influence of pH and lactic acid concentration on *Clostridium tyrobutyricum* during continuous growth in a pH-auxostat. J. Appl. Bacteriol..

[37] Lü F., Chen M., He P.J., Shao L.M. (2008). Effects of ammonia on acidogenesis of protein-rich organic wastes. Environ. Eng. Sci..

[38] Rezazadeh F., Kowsar R., Rafiee H., Riasi A. (2019). Fermentation of soybean meal improves growth performance and immune response of abruptly weaned Holstein calves during cold weather. Anim. Feed. Sci. Technol..

[39] Jones C.K., DeRouchey J.M., Nelssen J.L., Tokach M.D., Dritz S.S., Goodband R.D. (2010). Effects of fermented soybean meal and specialty animal protein sources on nursery pig performance. J. Anim. Sci..

[40] Bellés M., Del Mar Campo M., Roncalés P., Beltrán J.A. (2019). Supranutritional doses of vitamin E to improve lamb meat quality. Meat Sci..

[41] Wang Y., Wang Y., Wang B., Mei X., Jiang S., Li W. (2019). Protocatechuic acid improved growth performance, meat quality, and intestinal health of Chinese yellow-feathered broilers. Poult. Sci..

[42] Lee S.H., Choe J.H., Choi Y.M., Jung K.C., Rhee M.S., Hong K.C., Lee S.K., Ryu Y.C., Kim B.C. (2012). The influence of pork quality traits and muscle fiber characteristics on the eating quality of pork from various breeds. Meat Sci..

[43] Feng H.Y., Qu H., Liu Y., Shi Y.H., Wu S.L., Bao W.B. (2020). Effect of fermented soybean meal supplementation on some growth performance, blood chemical parameters, and fecal microflora of finishing pigs. Rev. Bras. Zootec.-Braz. J. Anim. Sci..

[44] Hao L.H., Su W.F., Zhang Y., Wang C., Xu B.C., Jiang Z.P., Wang F.Q., Wang Y.Z., Lu Z.Q. (2020). Effects of supplementing with fermented mixed feed on the performance and meat quality in finishing pigs. Anim. Feed. Sci. Technol..

[45] Klem T.B., Bleken E., Morberg H., Thoresen S.I., Framstad T. (2010). Hematologic and biochemical reference intervals for Norwegian crossbreed grower pigs. Vet. Clin. Pathol..

[46] Liu H., Ji H.F., Zhang D.Y., Wang S.X., Wang J., Shan D.C., Wang Y.M. (2015). Effects of *Lactobacillus* brevis preparation on growth performance, fecal microflora and serum profile in weaned pigs. Livest. Sci..

[47] Liu C., Chu D., Kalantar-Zadeh K., George J., Young H.A., Liu G. (2021). Cytokines: From Clinical Significance to Quantification. Adv. Sci..

[48] Avain A., Azad M.A., García Y., García Y., Martínez Y. (2024). Effects of Ganoderma lucidum Powder on the Growth Performance, Immune Organ Weights, Cecal Microbiology, Serum Immunoglobulins, and Tibia Minerals of Broiler Chickens. Vet. Sci..

[49] Jiang D., Yang M., Xu J., Deng L., Hu C., Zhang L., Sun Y., Jiang J., Lu L. (2023). Three-stage fermentation of the feed and the application on weaned piglets. Front. Vet. Sci..

[50] Yan H., Jin J.Q., Yang P., Yu B., He J., Mao X.B., Yu J., Chen D.W. (2022). Fermented soybean meal increases nutrient digestibility via the improvement of intestinal function, anti-oxidative capacity and immune function of weaned pigs. Animal.

[51] Cheng Z.J., Huang H., Zheng P., Xue M., Ma J., Zhan Z., Gan H., Zeng Y., Lin R., Li S. (2022). Humoral immune response of BBIBP COVID-19 vaccination before and after the booster immunization. Allergy.

[52] Gazzinelli-Guimarães A.C., Gazzinelli-Guimarães P.H., Nogueira D.S., Oliveira F.M., Barbosa F.S., Amorim C.C., Cardoso M.S., Kraemer L., Caliari M.V., Akamatsu M.A. (2018). IgG Induced by Vaccination with *Ascaris suum* Extracts Is Protective Against Infection. Front. Immunol..

[53] Wang J., Xiao Y., Li J., Qi M., Tan B. (2021). Serum biochemical parameters and amino acids metabolism are altered in piglets by early-weaning and proline and putrescine supplementations. Anim. Nutr..

[54] Duan J., Yin J., Ren W., Liu T., Cui Z., Huang X., Wu L., Kim S.W., Liu G., Wu X. (2016). Dietary supplementation with L-glutamate and L-aspartate alleviates oxidative stress in weaned piglets challenged with hydrogen peroxide. Amino Acids.

[55] Gęgotek A., Skrzydlewska E. (2019). Biological effect of protein modifications by lipid peroxidation products. Chem. Phys. Lipids.

[56] Czech A., Grela E.R., Nowakowicz-Dębek B., Wlazło Ł. (2021). The effects of a fermented rapeseed meal or/and soybean meal additive on the blood lipid profile and immune parameters of piglets and on minerals in their blood and bone. PLoS ONE.

[57] Gu X., Li Z., Wang J., Chen J., Jiang Q., Liu N., Liu X., Zhang F., Tan B., Li H. (2021). Fermented Cottonseed Meal as a Partial Replacement for Soybean Meal Could Improve the Growth Performance, Immunity and Antioxidant Properties, and Nutrient Digestibility by Altering the Gut Microbiota Profile of Weaned Piglets. Front. Microbiol..

[58] de Vos W.M., Tilg H., Van Hul M., Cani P.D. (2022). Gut microbiome and health: Mechanistic insights. Gut.

[59] Wang X., Zhang P., Zhang X. (2021). Probiotics Regulate Gut Microbiota: An Effective Method to Improve Immunity. Molecules.

[60] Szliszka E., Czuba Z.P., Domino M., Mazur B., Zydowicz G., Krol W. (2009). Ethanolic extract of propolis (EEP) enhances the apoptosis-inducing potential of TRAIL in cancer cells. Molecules.

[61] Fujisaka S., Watanabe Y., Tobe K. (2023). The gut microbiome: A core regulator of metabolism. J. Endocrinol..

[62] Barko P.C., McMichael M.A., Swanson K.S., Williams D.A. (2018). The Gastrointestinal Microbiome: A Review. J. Vet. Intern. Med..

[63] Shobharani P., Halami P.M. (2016). In vitro evaluation of the cholesterol-reducing ability of a potential probiotic *Bacillus* spp.. Ann. Microbiol..

[64] Kim K., Song M., Liu Y., Ji P. (2022). Enterotoxigenic *Escherichia coli* infection of weaned pigs: Intestinal challenges and nutritional intervention to enhance disease resistance. Front. Immunol..

[65] Dubreuil J.D. (2017). Enterotoxigenic *Escherichia coli* and probiotics in swine: What the bleep do we know?. Biosci. Microbiota Food Health.

[66] Niu T., Jiang Y., Fan S., Yang G., Shi C., Ye L., Wang C. (2022). Antiviral effects of *Pediococcus* acidilactici isolated from Tibetan mushroom and comparative genomic analysis. Front. Microbiol..

[67] Della Monica E., Kruasuwan W., Wankaew N., Arigul T., Wongsurawat T., Lazzi C., Gatti M., Levante A. (2026). Hybrid genome assembly and phenotypic assays reveal carbohydrate metabolism diversity in *Lacticaseibacillus* strains. Appl. Microbiol. Biotechnol..

[68] Gong G., Duojie A., He Y., Yang J., Zhang X., Chen Y., Zhu Y. (2025). Metabolism of prebiotic oligosaccharides by lactic acid bacteria isolated from rabbits: Insights into strain-specific utilization and applications. J. Appl. Microbiol..

[69] Luo R., Liu C., Li Y., Liu Q., Su X., Peng Q., Lei X., Li W., Menghe B., Bao Q. (2023). Comparative Genomics Analysis of Habitat Adaptation by *Lactobacillus* kefiranofaciens. Foods.

[70] Iarusso I., Mahony J., Pannella G., Lombardi S.J., Gagliardi R., Coppola F., Pellegrini M., Succi M., Tremonte P. (2025). Diversity of *Lactiplantibacillus plantarum* in Wild Fermented Food Niches. Foods.

[71] Johnson A., Miller E.A., Weber B., Figueroa C.F., Aguayo J.M., Johny A.K., Noll S., Brannon J., Kozlowicz B., Johnson T.J. (2023). Evidence of host specificity in *Lactobacillus* johnsonii genomes and its influence on probiotic potential in poultry. Poult. Sci..

[72] Lee J.Y., Han G.G., Kim E.B., Choi Y.J. (2017). Comparative genomics of *Lactobacillus salivarius* strains focusing on their host adaptation. Microbiol. Res..

